# Exploring the Blends’ Miscibility of a Novel Chitosan Derivative with Enhanced Antioxidant Properties; Prospects for 3D Printing Biomedical Applications

**DOI:** 10.3390/md21070370

**Published:** 2023-06-22

**Authors:** Georgia Michailidou, Alexandra Zamboulis, Dimitrios N. Bikiaris

**Affiliations:** Laboratory of Polymer and Colors Chemistry and Technology, Department of Chemistry, Aristotle University of Thessaloniki, 541 24 Thessaloniki, Greece; michailidougeorgia18@gmail.com (G.M.); azamboulis@gmail.com (A.Z.)

**Keywords:** chitosan derivative, polymer blends, miscibility, 3D printing

## Abstract

Chitosan is a polysaccharide vastly examined in polymer science for its unique structure. In the present study, CS was derivatized with 2-methoxy-4vinylphenol (MVP) in four different ratios through a free radical reaction. The CS-MVP derivatives were characterized through FTIR, 1H-NMR, XRD, swelling, and solubility measurements. Owing to the enhanced antioxidant character of the MVP monomer, the antioxidant activity of the CS-MVP derivatives was assessed. In the optimum CS-MVP ratio, blends between CS and CS-MVP were prepared in ratios of 90:10, 80:20, 70:30, 60:40, 50:50, 40:60, 30:70, 20:80, and 10:90 *w*/*w*, and their miscibility was examined by scanning electron microscopy (SEM) and viscosity measurements. In the optimum ratios, highly concentrated inks were prepared, and their viscosity measurements revealed the successful formation of highly viscous gels with shear thinning behavior. These inks could be appropriate candidates for biomedical and drug delivery applications.

## 1. Introduction

Chitosan (CS) is a natural polysaccharide derived from chitin through chemical or enzymatic deacetylation [1]. It is a non-toxic material with favorable properties, namely biocompatibility, biodegradability, mucoadhesion, and adsorption properties [2,3,4,5]. Amino and hydroxylic groups are present on the structure of CS, rendering CS modification feasible. In the literature, modification of CS structures with various monomers has been conducted, including the addition of carboxylic, sulfonic, and acrylic groups, as well as the addition of unsaturated monomers [6,7]. The addition of these groups onto the CS backbone alters the characteristics of the polysaccharide since the derivatives could potentially present ameliorated swelling, solubility, or antioxidant properties. 

For the enhancement of the final properties of CS materials, the preparation of blends with various polymers is a commonly studied technique. Overall, the blending of polymers provides versatile options to develop materials with enhanced properties, functionalities, or activities. The methods for the preparation of polymeric blends are solution blending and melt mixing; however, CS blends are mainly prepared through solution mixing with other polymers, including both natural and synthetic polymers [8,9]. Currently, CS has been recently examined as a material for 3D printing applications owed to its ability to form hydrogels with shear thinning behavior [10]. CS structure has been modified with various monomers to improve the viscosity, as well as the printability of the final inks [11,12]. 

Among the various monomers utilized for the modification of CS structure, phenolic monomers, such as hydroxy- and dihydroxymethybenzoate, have been examined, identifying materials with broad antimicrobial spectra [13]. The compound 2-methoxy-4vinylphenol (MVP) is an aromatic compound derived from buckwheat. It has a pleasant flavor and has been employed as a flavoring agent. Owing to its structure, it presents antioxidant, antimicrobial, anti-inflammatory, and anticancer properties [14,15,16]. Due to the presence of an unsaturated bond in the MVP structure, many research groups have investigated the synthesis of its homopolymer and copolymers with acrylic derivatives through radical polymerization [17,18]. The homopolymer was easily synthesized via mild conditions through radical polymerization, developing composite materials with carbon nanotubes with enhanced stability. Furthermore, the obtained copolymers were oil and water repellent, simultaneously forming hydrogen bonds to the hydroxyapatite of teeth.

**Figure 1:**
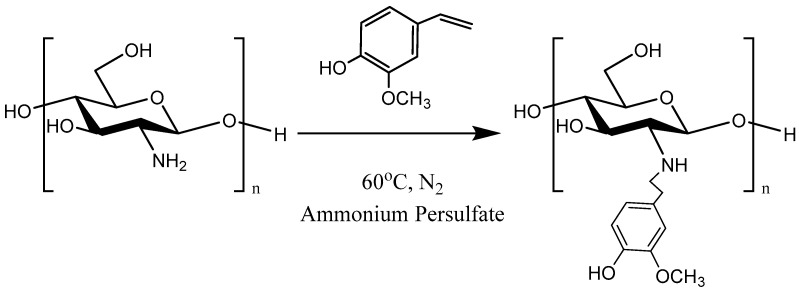
Schematic reaction of CS with MVP.

In the present study, the synthesis of the CS-MVP derivative was implemented for the first time (Figure 1). Four different substitution degrees were studied, and the obtained derivatives were characterized. The optimal CS-MVP derivative was further used for the preparation of CS/CS-MVP blends for printing inks. The miscibility of CS and CS-MVP was initially examined (CS/CS-MVP ratios 90:10, 80:20, 70:30, 60:40, 50:50, 40:60, 30:70, 20:80 and 10:90). In the optimum ratios, CS/CS-MVP concentrated inks were synthesized and their viscosity values, along with their potential application for 3D bioprinting, were assessed. The materials are exceptional candidates for drug delivery applications. 

## 2. Results and Discussion

### 2.1. CS-MVP Derivatives

One of the primary aims of the present study was the preparation of CS derivatives with MVP monomer, an aromatic compound extensively used as a flavoring agent [14,19,20]. In general, the presence of monomers on the CS backbone greatly affects the final characteristics of CS derivatives [21]. In our study, modification with MVP aimed to confer antioxidant properties to CS. CS modification was investigated with four different CS/MVP molar ratios: 4:1, 2:1, 1:1, and 1:2. CS modification was performed via a radical reaction according to Figure 1.

**Figure 2:**
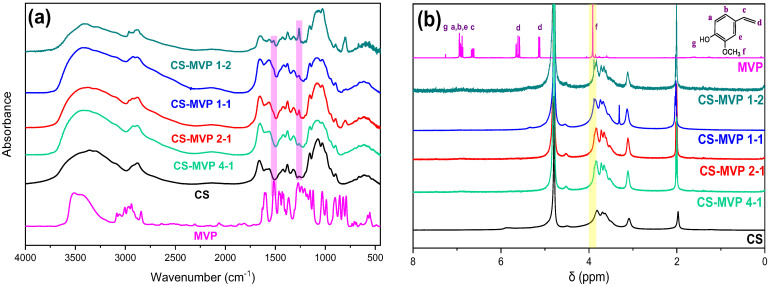
(**a**) FTIR spectra and (**b**) 1H-NMR spectra of CS, MVP, and CS-MPV derivatives.

To verify the successful preparation of CS-MVP derivatives, FTIR spectra were recorded (Figure 2a). Neat CS presents characteristic peaks throughout its spectrum. A broad band at 3000–3600 cm^−1^ corresponds to the overlapped stretching of N-H and O-H bonds of the characteristic amino and hydroxyl groups. Peaks at 1645 cm^−1^ and 1567 cm^−1^ are attributed to the vibration of C=O and N-H of amides I and II, correspondingly. Further peaks at 1423 cm^−1^, 1375 cm^−1^, 1153 cm^−1^, and 1066 cm^−1^ are ascribed to the CH_2_ bending, CH_3_ symmetrical deformations, asymmetric stretching of the C-O-C bridge, and C-O stretching, respectively [22]. The signals of MVP appear at 3515 cm^−1^ for the O-H stretching vibration of the hydroxyl groups of the phenolic ring; and at 1602 cm^−1^ and 1513 cm^−1^, corresponding to the vibration of the aromatic ring; while at 1426 cm^−1^, the vinyl group of MVP is observed. Regarding the spectra of the CS-MVP derivatives, the formation of a covalent bond between the amino groups of CS and MVP is confirmed by the peak at 1261 cm^−1^, corresponding to C-N amine stretching. Moreover, by increasing the MVP ratio, the intensity of the aforementioned peak increased. Furthermore, a peak at 1516 cm^−1^ was detected, attributed to the vibration of the aromatic ring. Additionally, the characteristic broad curve of CS present at 3350 cm^−1^ shifted to 3448 cm^−1^ in the CS-MVP spectra owing to the formation of hydrogen bonds between the CS free functional groups and the MVP characteristic groups. For further confirmation of the successful derivatization of CS, 1H-NMR spectra were acquired (Figure 2b). In the MVP spectrum, the protons of the vinyl groups Hc and Hd were observed at 6.6, 5.1, and 5.6 ppm, respectively. Protons Ha, Hb and He of the phenolic ring gave rise to resonance signals at 6.8–7.0 ppm. Finally, the Hf protons of the methoxy groups were observed at 3.9 ppm. In the CS-MVP derivative, confirmation of the successful derivatization was obtained through the latter protons. Indeed, the resonance signal of increasing intensity with increasing MVP ratio, at 3.8 ppm, validated the successful synthesis of CS-MVP. Furthermore, according to the literature, the absence of the vinyl protons in the derivatives further confirms the formation of a covalent bond between CS and MVP [23]. The intense peak at 4.79 ppm present in all spectra can be ascribed to the deuterated water used to dissolve the compounds. 

Table 1 summarizes the degree of substitution (DS) measured by titration for the four derivatives. The results show that the 4:1 ratio has a DS equal to 10.2%, while as expected, DS increases when the reaction is conducted by increasing MVP quantities. The highest obtained DS is 15.4% for sample CS-MVP 1-2. 

**Figure 3:**
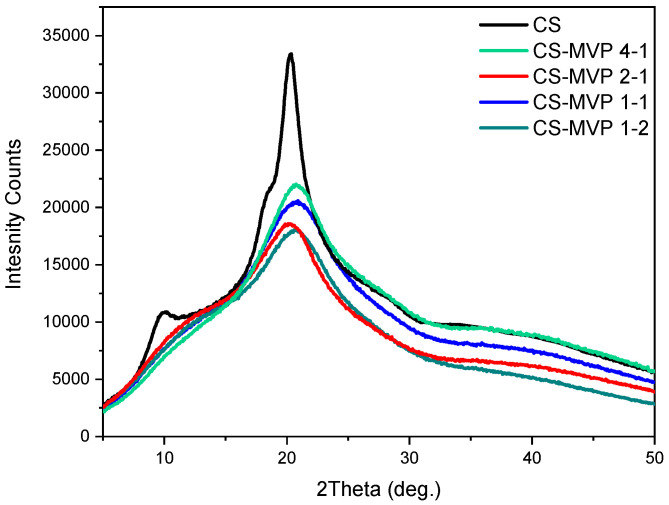
XRD diffractograms of CS, MVP, and CS-MPV derivatives.

The crystalline phase of the CS-MVP derivatives was further examined through XRD measurements. CS is a semicrystalline polysaccharide with two characteristic peaks at 10° and 20° [24]. Concerning the CS-MVP derivatives, they appear as semicrystalline materials (Figure 3). The characteristic peaks of CS appeared broader, and they shifted to higher 2θ values. This alteration of the XRD patterns in comparison to CS is attributed to the intercalation of the molecule of MVP between the CS chains. Indeed, the amino groups of CS that are covalently bonded to the MVP moiety are unable to form hydrogen bonds. Moreover, the aromatic ring of MVP provokes some steric hindrance, preventing the folding of CS-MVP chains. According to the literature, this behavior is typical for CS derivatives [25]. 

**Figure 4:**
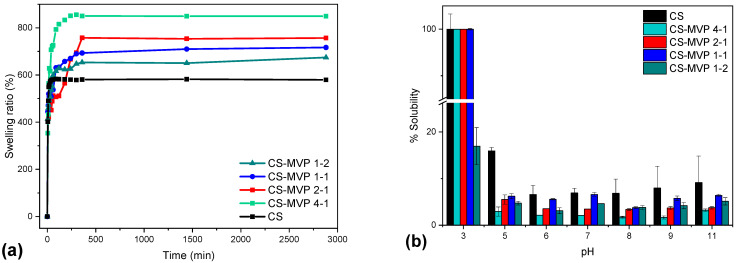
Measurements of CS and CS-MPV samples in ratios of 4:1, 2:1, 1:1, and 1:2: (**a**) swelling ability at a pH of 7.4; and (**b**) solubility at pH values of 3, 5, 6, 7, 8, 9, and 11.

The swelling ability of the materials designed for pharmaceutical and engineering purposes is a crucial factor affecting the properties of the final products. The swelling ability of neat CS has already been examined by our group, as well as many others. Neat CS is able to swell up to 300–600% of its dry mass at 25 °C [26,27]. Figure 4a presents the swelling ability of the materials CS-MVP 4-1, 2-1, 1-1, and 1-2. The materials CS-MVP presented ameliorated swelling ability for each ratio examined in comparison to CS. Not surprisingly, the MVP content affects the swelling ability of the polysaccharides. CS-MVP 4-1 exhibited the highest swelling ability, equal to 850% of its dry mass, while CS-MVP 1-2 had the lowest ability, equal to 650% of its dry mass. It is obvious that the increase in the percentage of MVP in the CS-MVP backbone results in decreased swelling ability of the final polymers. 

The addition of hydrophilic functional groups on the CS backbone results in increased swelling ability of the CS derivatives [21]. The structure of MVP consists of a hydroxyl group and a methoxy group. The modification of the CS backbone with hydroxylic groups ameliorates the swelling ability. In addition to the OH groups, the methoxy groups are also able to interact with H-bond donors [28]. Consequently, an increase in the swelling behavior was expected by increasing the degree of MVP substitution. However, the opposite trend was observed: a decrease in the swelling ability by increasing the MVP ratio (Figure 4). This behavior is attributed to the highly hydrophobic aromatic ring. By increasing the MVP ratio, non-polar interactions (e.g., π-π interactions) occur among the aromatic rings, leading to decreased swelling ability of the materials in aqueous solutions [29]. 

In a further step, the solubility and hydrophilicity of the CS derivatives were examined (Figure 4a,b). CS is a soluble polymer in an acidic pH but remains insoluble in neutral and alkaline environments [30]. This behavior arises from its chemical structure. The amino groups are considered as bases with a pKa value of 6.3. At pH < 6 (less than pKa), the amino groups are protonated, and the polymer is soluble. As the pH increases to greater than 6, the amino groups are deprotonated, rendering the polymer insoluble [31]. 

Concerning the results of Figure 4b, the CS-MVP 4-1, 2-1, and 1-1 derivatives remained soluble at pH = 3. By increasing the pH value to 5, the solubility of the derivatives diminished since the deprotonation of the free amino groups on the CS-MVP structure occurred. However, a further increase in pH resulted to increased solubility of the derivatives. This behavior could be associated with possible chain scission of CS by the presence of NaOH solution, leading to lower molecular weight [32]. Moreover, by increasing the MVP substitution of the CS-MVP derivatives, increased solubility in any pH value was acquired. 

Regarding the derivative CS-MVP 1-2, at a pH of 3, lower solubility was detected compared to the CS-MVP ratios of 4:1, 2:1, and 1:1, at which it was equal to 18%. When the pH value is equal to 5, the solubility decreases, although a further increase in the pH value affects to a lesser extent the solubility of the material. 

The solubility results are in accordance with contact angle measurements (Table 2). Large θ values reveal higher hydrophobicity of the material, whereas small θ angles indicate high hydrophilicity [33]. CS has a high contact angle equal to 74.5°, revealing its low hydrophilicity. According to Table 2, increasing the substitution of the CS backbone resulted in higher hydrophilicity of the CS-MVP derivatives with contact angle measurements equal to 73.5°, 67.8°, and 58° for the ratios of 4:1, 2:1, and 1:1, respectively. Through the addition of MVP to CS structure, additional functional groups are available for the formation of hydrogen bonds with water, rendering the derivatives more soluble, along with having higher hydrophilicity. However, the theta of the CS-MVP 1-2 derivative was equal to 82.6°, which was higher than that of neat CS. Consequently, the extensive increased presence of the hydrophobic phenolic ring of MVP in the CS backbone enhanced the hydrophobicity of the material and simultaneously diminished its solubility. 

**Figure 5:**
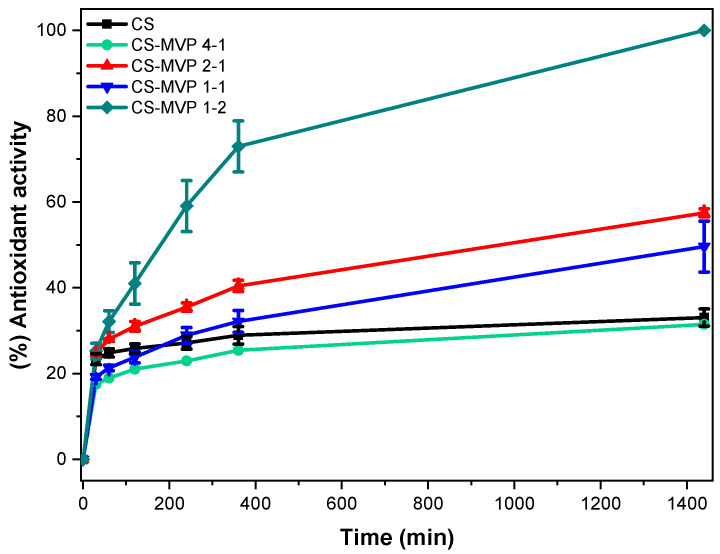
Antioxidant activity of the samples CS, CS-MVP 4-1, CS-MVP 2-1, CS-MVP 1-1, and CS-MVP 1-2.

The intrinsic antioxidant character of CS has already been examined [34]; so have the antioxidant properties of MVP, which have been attributed to the phenolic hydroxyl group [16]. Figure 5 presents the antioxidant activity of CS and CS-MVP materials. With the exception of CS-MVP 4-1, the presence of the MVP monomer resulted in amelioration of the antioxidant activity of CS. The accuracy of the measurements is evident by the narrow error bars corresponding to each sample. For CS-MVP 4-1, a slight decrease in the antioxidant activity compared to neat CS was detected. CS-MVP 1-2 had the best antioxidant activity, with 73% inhibition in 6 h, while after 24 h, the inhibition reached 99%. CS-MVP 1-2 exhibited the highest degree of substitution, resulting in ameliorated antioxidant activity. Samples 2-1 and 1-1 had activity equal to 57% and 50% after 24 h, respectively. For samples CS-MVP 2-1 and 1-1, the swelling ability of the materials affected the antioxidant activity. Sample CS-MVP 2-1 had a higher swelling ability than sample 1-1. Therefore, the DPPH radicals are able to interact to a greater extent with the polymers, thus presenting improved antioxidant activity. This correlation of the antioxidant activity of a material with its swelling ability has already been reported in the literature with carboxymethyl cellulose, whey protein, and pectin [35]. 

### 2.2. CS/CS-MVP Blends

Based on the satisfactory swelling, solubility, and antioxidant activity, CS-MVP 2-1 was further used for the preparation of blends with neat CS. The CS/CS-MVP blends were prepared in 90:10, 80:20, 70:30, 60:40, 50:50, 40:60, 30:70, 20:80, and 10:90 mass ratios, while the neat materials CS and CS-MVP are referred to as CS/CS-MVP 100/0 and CS/CS-MVP 0/100, respectively. 

**Figure 6:**
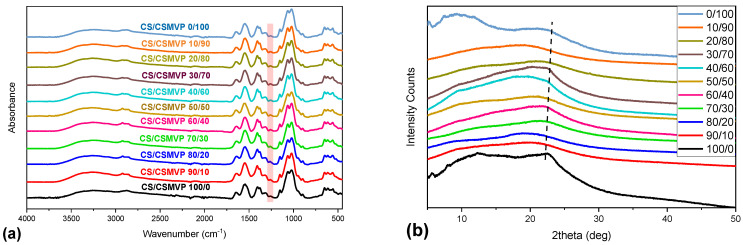
(**a**) FTIR spectra; and (**b**) XRD diffractogram of the CS/CSMVP blends.

The interactions occurring between CS and CS-MVP were examined through ATR measurements. Figure 6a presents the spectra of CS/CS-MVP blends. By increasing the percentage of CS-MVP in the blends, the broad peak corresponding to the vibration of O-H and N-H bonds was slightly shifted. Moreover, the characteristic peak of the CS-MVP material present at 1261 cm^−1^, corresponding to the vibration of the C-N bond, is present in the spectra of all the CS/CS-MVP blends. As expected, the increase in CS-MVP content in the blend resulted in an increase in the intensity of the peak, corresponding to the C-N bond. 

In a further step, the crystallinity of the blends was examined through XRD measurements (Figure 6b). As already discussed, both CS and CS-MVP materials are semi-crystalline with distinct characteristic peaks in their diffractograms. The CS/CS-MVP blends are also semi-crystalline materials. The broad peak present at 22.5° for the 100:0 ratio shifted to higher 2θ values by increasing the CS-MVP content of the blends. This behavior was observed up to sample CS/CS-MVP 20/80, while the 90:10 ratio revealed a broad peak at 20° The alteration of the characteristic peaks of the polymeric materials is characteristic when CS interacts with other polymers through hydrogen bonds. This interaction results in the elimination of the peak at 10° and decreases of the intensity of the peak at 20° [36].

The miscibility of polymeric materials can be assessed through various techniques, including FTIR measurements, SEM images, DSC analysis, and viscosity measurements [37]. In the context of the present study, the miscibility of the CS and CS-MVP materials was examined via SEM images and viscosity measurements. 

**Figure 7:**
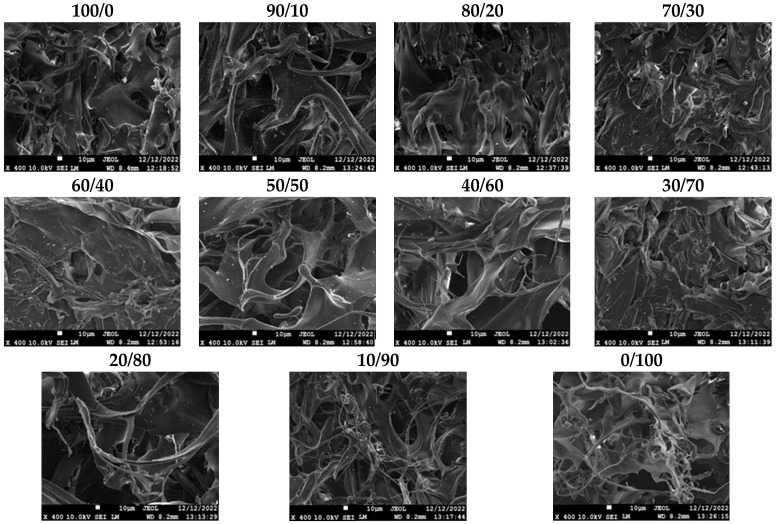
SEM images of the CS/CSMVP blends in ratios of 100:0, 90:10, 80:20, 70:30, 60:40, 50:50, 40:60, 30:70, 20:80, 10:90, and 0:100.

The surfaces of the lyophilized CS/CS-MVP blends are depicted in Figure 7. Neat CS (material 100/0) has an irregular morphology with many flattened surfaces, while CS-MVP (picture 0/100) is more fibrous. Blends 10/90 and 20/80 tend to be similar to the 0/100 structure. Likewise, blends 90/10 and 80/20 present a morphology that resembles sample 100/0, with irregular and flattened surfaces. The intermediate ratios 70:30, 60:40, 50:50, 40:60, and 30:70 behave as blends and present more solid surfaces in their morphology, along with slightly fibrous areas. 

Detection of interphase or spherical shapes would indicate immiscible polymeric materials [38]. None of the blends presented any signs of interphases; therefore, all blends are completely miscible.

**Figure 8:**
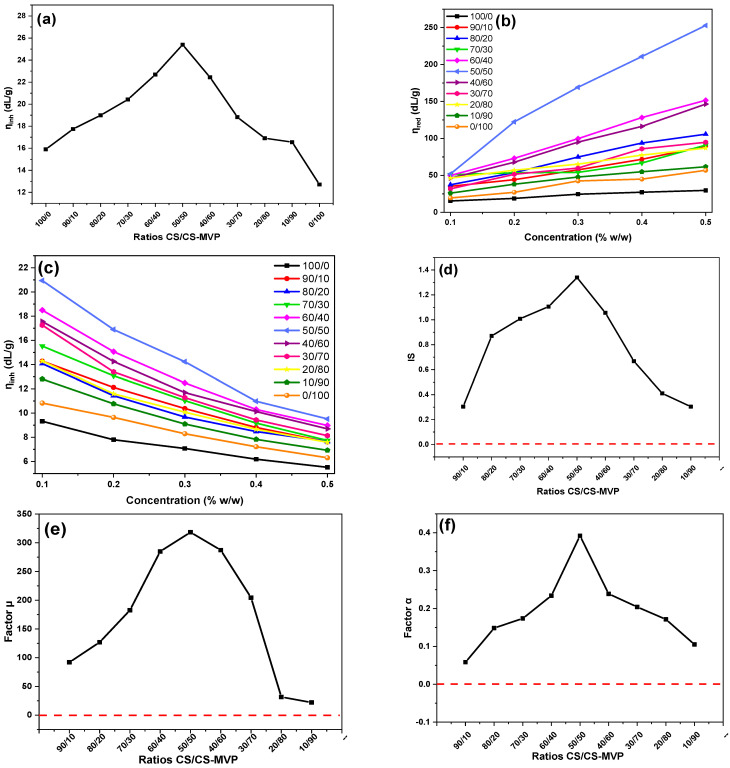
(**a**) Intrinsic viscosity values; (**b**) reduced viscosity values toward concentration; (**c**) inherent viscosity values toward concentration; (**d**) synergic interaction index (IS) (red line indicates the minimum value required for viscous synergy); (**e**) miscibility parameter μ; (**f**) miscibility parameter a of CS/CSMVP blends toward concentration (the red lines indicate the minimum values required for miscible blends).

SEM images provide an indication concerning the miscibility of CS/CS-MVP blends. For further confirmation, viscosity measurements were obtained via an Ostwald viscometer, and the miscibility of the CS/CS-MVP blends was examined according to the works of Chee et al. [39] and Sun et al. [40]. Figure 8a presents the intrinsic viscosity values of the CS/CS-MVP blends for ratios of 90:10–10:90. The viscosity value of neat CS-MVP derivative is lower than that of neat CS. However, the addition of 10% of CS solution resulted in an increased viscosity value compared to CS. This behavior confirms the strong interaction between the two polymers. Higher viscosity values than neat CS and CS-MVP were detected in all other CS/CS-MVP ratios. As shown in Figure 8a, the highest viscosity values were obtained with the ratios of 60:40, 50:50, and 40:60. In Figure 8b, the increase in the reduced viscosity by increasing the polymeric concentration is shown, whereas Figure 8c depicts the reduction in the inherent viscosity with the increase in the concentration. This behavior is typical for dilute CS solutions [41,42]. Through the viscosity measurements of dilute polymeric solutions, the behavior of higher concentrated solutions can be estimated [40]. For all CS/CS-MVP blends, the obtained viscosity values are higher compared to the initial polymers. The increased viscosity is typical for miscible blends with viscous synergy. 

Furthermore, the synergy of the components of the blends was calculated according to Equation (4). For all ratios, a positive value of the IS factor was obtained, indicating the viscous synergy of the system (Figure 8d). Optimum interactions leading to enhanced synergy were observed for the ratios showing the highest viscosity values, i.e., 60:40, 50:50, and 40:60. Additionally, miscibility factors μ and α were also calculated according to Equations (9) and (10), respectively, and are depicted in Figure 8e,f. The positive values of the μ and α factors prove the miscibility of the blends in all the studied ratios. Furthermore, the strong interactions between the CS and CS-MVP polymers in the ratios of 60:40, 50:50, and 40:60 are also evident in Figure 8e,f. 

### 2.3. CS/CS-MVP Inks

**Figure 9:**
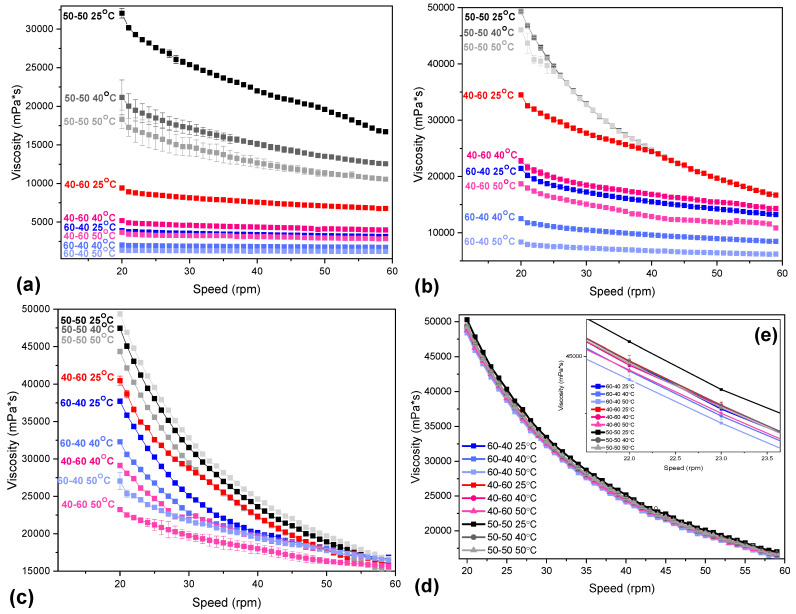
Viscosity dependency over increasing rotational speed of 20–60 rpm for samples CS/CSMVP 60/40, 50/50, and 40/60 in concentrations of: (**a**) 4% *w*/*v*; (**b**) 5% *w*/*v*; (**c**) 6% *w*/*v*; and (**d**) 7% *w*/*v*; and (**e**) viscosity dependency over increasing rotational speed of 21–24 rpm of samples CS/CSMVP 60/40, 50/50, and 40/60 in concentration of 7% *w/v* at temperatures 25 °C, 40 °C, and 50 °C.

CS blends have already been examined by our group for drug delivery of chloramphenicol [27] and fluticasone propionate, and their application in 3D printing has already been established [11,43]. Within this scope, concentrated inks of high viscosity were formulated with the CS/CS-MVP blends, and their potential application in pneumatic 3D extrusion was examined. The IS factor calculated through miscibility studies indicated a high viscous synergy for the 60:40, 50:50, and 40:60 CS/CS-MVP blends, and in these ratios, inks were prepared in concentrations of 4% *w*/*v*, 5% *w*/*v*, 6% *w*/*v*, and 7% *w*/*v*. Their viscosity behavior was assessed at increasing temperatures and rotational speeds (Figure 9). 

The viscosity of a polymeric solution is affected by various factors, including polymer concentration, molecular weight, utilized solvent, and temperature [44]. The CS/CS-MVP inks in every ratio and concentration exhibited a shear-thinning behavior since the viscosity decreased by increasing the applied rotational speed. An exception was sample CS/CS-MVP 60/40 4% *w*/*v,* which at higher temperature values remained unaffected by increasing the rotational speed. Furthermore, increasing the temperature resulted in a decrease in the viscosity of the polymeric blends, attributed to the increased mobility of both the polymeric chains and the solvent molecules. According to Brugnerotto et al. [45], the mobility of the polymeric chains increases with increased applied temperature, leading to hydrogels with inferior mechanical properties. Viscosity diagrams in Figure 9 revealed that the viscosity of highly viscous materials is affected to a lesser extent by the increase in temperature. This outcome is evident in the viscosity values of the samples in Figure 9d,e, in which the viscosity of the CS/CS-MVP samples was scarcely affected by the increase in the temperature. For the 4% *w/v* concentration, the decrease in the viscosity value of the 50:50 sample was equal to 140 mPa∙s for 20 rpm, while the reduction in the viscosity values of the 60:40 and 40:60 samples is equal to 27 mPa∙s and 56 mPa∙s, respectively. In contrast, regarding the 7% *w/v* concentration, the reduction in the viscosity was smaller (10, 6, and 9 mPa∙s for the ratios of 50:50, 40:60, and 60:40, respectively).

Moreover, the ratio of the polymeric blends affects the viscosity of the final inks. The 50/50 sample presented the greater viscosity than the 40/60 and 60/40 blends, indicating the strong interactions between the components of the blends, a result in accordance with the IS factor. Along with the ratio of the blends, the polymeric concentration greatly affects the viscosity of the solution. In their studies, Joohyang et al. [46] and Desbrieres [47] described a linear correlation between the concentration of a CS derivative and its viscosity. In the latter work, a Newtonian behavior of CS solutions up to 4.72 g/L was reported, while for higher concentrations, a non-Newtonian behavior was established. This behavior was detected in the CS/CS-MVP blends. The inks present a non-Newtonian behavior at high viscosity, while at low viscosities, the samples behave almost as Newtonian materials. 

The inks in general presented viscosity values ranging between 50 and 520 mPa∙s for an applied rotational speed of 20 rpm. According to the literature, inks designed for pneumatic extrusion require viscosity values ranging between 0.3 and 30 Pa s. Many groups have examined viscous gels of natural polysaccharides, namely CS/collagen blends with viscosity values of 500–800 mPa∙s [48] or sodium alginate, hyaluronic acid, and silk fibroin with viscosities ranging between 500 and 900 mPa∙s [49]. For the present CS/CS-MVP samples, the 6% *w/v* and 7% *w/v* concentrations were within the appropriate ranges for uniform extrusion. Consequently, the CS/CS-MVP inks are promising candidates for the formulation of inks for pneumatic extrusion 3D printing. 

## 3. Materials and Methods

### 3.1. Materials

CS with high molecular weight (310,000–375,000 Da) and a degree of deacetylation >75% and 2-methoxy-4-vinylphenol (MVP) with 99% purity were supplied by Sigma Aldrich Co. (St. Louis, MO, USA). All other utilized reagents were of analytical grade. 

### 3.2. Synthesis of CS-MVP

For the preparation of CS grafted with MVP derivative, 1 g of CS was initially dissolved in 150 mL of2% *v/v* aqueous acetic acid solution, forming a polymeric solution of 0.6% *w*/*v*. Proper amounts of MVP monomer were added to the CS solution (Table 3), and the ammonium persulfate initiator was added in a 0.2% *w*/*w* ratio vs. CS dry mass. The grafting reaction was conducted at 70 °C for 2 h under a nitrogen atmosphere. The mole ratios examined were CS/MVP 4:1, 2:1, 1:1, and 1:2. Then, the CS derivative was freeze-dried under reduced pressure at −60 °C. The product was purified using a Soxhlet apparatus with methanol to remove the remaining impurities. 

### 3.3. Preparation of CS/CS-MVP Blends

CS/CS-MVP blends were prepared by mixing CS and CS-MVP 2-1 at the following weight ratios: 90:10, 80:20, 70:30, 60:40, 50:50, 40:60, 30:70, 20:80, and 10:90. Briefly, separate solutions of CS and CS-MVP were prepared in 2% *v/v* acetic acid and blended under magnetic stirring. After overnight stirring, the solutions were centrifuged (4000 rpm, 5 min, Unicen 21, Orto alresa, Madrid, Spain) to remove the bubbles generated by stirring. Each blend was prepared in five concentrations of 0.1, 0.2, 0.3, 0.4, and 0.5% *w*/*v*. 

### 3.4. Preparation of CS/MVP Inks

The CS/CS-MVP inks were prepared at weight ratios of 60:40, 50:50, and 40:60. CS and CS-MVP materials were mixed as dry powders, and 2% *v/v* acetic acid solution was added. The solutions were stirred for 48 h, forming gels with concentrations of 4% *w*/*v*, 5% *w*/*v*, 6% *w*/*v*, and 7% *w*/*v*. The inks were centrifuged at 4000 rpm for 20 min for the removal of the air bubbles trapped during the stirring process. 

### 3.5. Characterization Techniques

#### 3.5.1. Fourier-Transformed Infrared Spectroscopies

The FTIR spectra of the samples were obtained by an FTIR spectrometer (model FTIR-2000, Perkin Elmer, Waltham, MA, USA). Briefly, a small amount of each sample was triturated with a proper amount of potassium bromide (KBr), and disks were formed under pressure. The spectra were collected in the range from 400 to 4000 cm^−1^ at a resolution of 4 cm^−1^ using 16 coadded scans, while the baseline was corrected and converted into absorbance mode.

ATR spectra of the samples were recorded using an IRTracer-100 (Shimadzu, Kyoto, Japan) equipped with a QATR™ 10 Single-Reflection ATR Accessory with a Diamond Crystal. The spectra were collected in the range from 450 to 4000 cm^−1^ at a resolution of 2 cm^−1^ (a total of 16 co-added scans), while the baseline was corrected and converted into absorbance mode.

#### 3.5.2. Nuclear Magnetic Resonance (1H-NMR) 

1H-NMR spectra were recorded in a deuterated aqueous solution of 2% acetic acid *v*/*v*. An Agilent 500 spectrometer was utilized (Agilent Technologies, Santa Clara, CA, USA) at room temperature. Spectra were internally referenced with tetramethylsilane (TMS) and calibrated using the residual solvent peaks.

#### 3.5.3. Wide-Angle X-ray Scattering (XRD)

X-ray diffraction (XRD) patterns were reported using an XRD-diffractometer (Rigaku-Miniflex 600, Chalgrove, Oxford, UK) with CuKα radiation for crystalline phase identification (λ = 0.15405 nm). All samples were scanned over the range of 2θ from 5° to 50° through a scan speed of 1°/min.

#### 3.5.4. Scanning Electron Microscopy (SEM) 

Scanning electron microscopy (SEM) images were obtained with a JEOL 2011 electron microscope (Akishima, Tokyo, Japan). The samples were placed in a holder and coated with carbon to administer a satisfying conductivity of the electron beam. Operating conditions were set at expediting voltage of 20 kV, probe current of 45 nA, and counting time of 60 s.

#### 3.5.5. Degree of Substitution

The degree of substitution of CS-MVP was calculated indirectly by measuring the numbers of CS’s and CS-MVP’s free amino groups according to the titration method described by Czechowska-Biskup et al. [50]. Briefly, 0.2 g of CS or CS-MVP were dissolved in 20 mL of HCl 0.1 M, and 10 mL of distilled water were added. Titration was performed with NaOH 0.1 M solution under continuous magnetic stirring. The volume of the added NaOH and the pH values were recorded until the pH of the solution reached a value of 3. Three replicates were performed for each sample.

#### 3.5.6. Contact Angle

For the calculation of the contact angle, films of approximately 2 × 2 cm^2^, prepared by solvent evaporation of 1% *w/v* polymeric solutions at 50 °C, were placed onto the microscope glass. Contact angles were measured in water, employing the sessile drop method with an Ossila Contact Angle Goniometer L2004A1 (Ossila Ltd., Shiefield, UK). The experiment was performed in triplicate. The results were expressed as means ± standard deviations (SDs).

#### 3.5.7. Swelling and Water Content Capacity

The swelling ability of the materials was evaluated by measuring the amount of water sorption aptitude of simulated body fluid (SBF) buffer (pH = 7.4). Each dry sample was carefully weighed (Wd) and immersed in SBF. The samples were then placed on filter paper to remove the excess surface water, and their weight (Wf) was measured at predetermined times (5 min, 10 min, 20 min, 30 min, 1 h, 2 h, 3 h, and 48 h). The swelling ratio and water content were calculated according to Equations (1) and (2), respectively.

Swelling ratio% = (Wf − Wd) × 100/Wd(1)

Water content% = (Wf − Wd) × 100/Wf(2)

#### 3.5.8. Solubility Measurements

The solubility of the CS-MVP derivatives was examined at different pH values. Approximately 20 mg of each material were added to 1 mL of solutions with different pH values. The examined pH values were 3, 5, 6, 7, 8, 9, and 11, and pH was adjusted with acetic acid solution 2% *v/v* for the acidic pH values and with NaOH 1 M solution for the basic solutions. The samples were stirred for 24 h and then centrifuged at 10,000 rpm. The undissolved material was dried and accurately weighed.

#### 3.5.9. Viscosity Measurements 

The intrinsic viscosity [η] of CS, CS-MVP, and CS/CS-MVP blends was measured using an Ubbelohde viscometer at 25 °C in a CH_3_COOH 2% *v/v* solution. 

The viscosity measurements of the CS/CS-MVP inks were performed at three different temperatures, namely 25 °C, 40 °C, and 50 °C, together with increasing rotational speed (20–60 rpm) using the SC29 spindle of a rheometer (BGD 157/TS, Biuged Instruments, Guangzhou, China).

#### 3.5.10. Viscous Synergy

The viscous synergy of the prepared CS/CS-MVP blends was calculated according to Pradhan and Roy [51]. The experimental viscosity of the blends (ηexp) was compared to the viscosity calculated theoretically (ηcalc) in the absence of interactions, according to Equation (3).

(3) $$\eta_{\mathrm{calc}}=x_{A}\eta_{A}+x_{B}\eta_{B}$$

x_A_ and x_B_ are the mole fractions of the components of the blends, while η_A_ and η_B_ are the intrinsic viscosities of the pure components A and B, respectively. Furthermore, the synergic interaction index (IS) was calculated according to Equation (4)

(4) $$\mathrm{IS}=\left(\eta_{\exp}–\eta_{\mathrm{calc}}\right)/\eta_{\mathrm{calc}}$$

If IS < 0, antagonistic interactions occur; if IS = 0, lack of interactions between the components is observed; and if IS > 0, viscous synergy is detected between the blends’ components. 

#### 3.5.11. Miscibility Study

In the present study, the miscible nature of the CS/CS-MVP blends was evaluated by viscometry according to the method suggested by Chee et al. [39]. According to this method, when two polymers are mixed in different weight ratios w_1_ and w_2_, their intermolecular interactions are defined by the differential interaction parameter ΔB, which is calculated according to the Equation (5):

(5) $$\Delta B=\frac{b-\;\bar{b}}{2w_{1}w_{2}}$$

where $\bar{b}$ is calculated from Equation (6)

(6) $$\;\bar{b}=w_{1}b_{11}+w_{2}b_{22}$$

b_11_ and b_22_ are the slopes of the reduced viscosity curves of the pure components, while b is related to Huggins’ coefficient kH according to Equation (7):

b = k_H_[η]^2^ (7)

For a blend composed of two polymeric materials, b is calculated according to Equation (8)

b = w_1_^2^b_11_ + w_2_^2^b_22_ + 2w_1_w_2_b_12_ (8)

b_12_ is the slope of the reduced viscosity curve for the blend solution. However, Chee suggested a more effective interaction parameter μ aimed at eliminating possible errors when [η]_1_ and [η]_2_ are different, defined by Equation (9).

(9) $$\mu \;=\frac{\Delta B}{{\left({\left[\eta \right]}_{2}-{\left[\eta \right]}_{1}\right)}^{2}}$$

[η]_1_ and [η]_2_ are the intrinsic viscosities of pure components CS and CS-MVP, respectively. If μ ≥ 0, the blend is referred to as miscible, while if μ < 0, then the examined blend is characterized as immiscible. Following the work of Chee, Sun et al. [40] suggested the interaction parameter α for the investigation of the miscibility of a blend, derived from Equation (10).

(10) $$\alpha \;=\;\mathrm{Km}\;-\frac{K_{1}{\left[\eta \right]}_{1}^{2}w_{1}^{2}+K_{2}{\left[\eta \right]}_{2}^{2}w_{2}^{2}+2\sqrt{K_{1}K_{2}{\left[\eta \right]}_{1}{\left[\eta \right]}_{2}w_{1}w_{2}}}{{\left({\left[\eta \right]}_{1}w_{1}+{\left[\eta \right]}_{2}w_{2}\right)}^{2}}$$

K_1_ and K_2_ are the Huggins’ constants for the pure components 1 and 2, respectively, and Km refers to the blend. If α ≥ 0, the blend is miscible, while if a < 0, then it is referred to as immiscible.

#### 3.5.12. Antioxidant Activity

The antioxidant activity of the samples was determined according to the 2,2-diphenyl-1-picrylhydrazyl (DPPH) method. In detail, 100 mg of the CS and CS-MVP sponges were added to a 0.1 mM methanol DPPH solution. A DPPH/MeOH solution was applied as a reference sample. The samples were kept in a closed chamber in the absence of UV light, and their absorption was calculated with the aid of a UV-Vis spectrometer (UV Probe 1650, Shimadzu, Tokyo, Japan) at 517 nm at 30 min, 1 h, 2 h, 4 h, 6 h, and 24 h. The free radical scavenging activity was calculated using Equation (11)

(11) $$\mathrm{Free}\;\mathrm{radical}\;\mathrm{scavenging}\;\mathrm{activity}\;\left(\%\right)=\frac{Absorbance\;of\;control-Absorbance\;of\;sample}{Absorbance\;of\;control}\;\times \;100$$

## 4. Conclusions

In the present study, CS was modified with MVP through a free radical reaction in four different ratios. The successful formation of the CS-MVP derivatives was confirmed by FTIR and NMR spectroscopies. The new materials present enhanced swelling and antioxidant properties compared to neat CS. Miscible viscous blends of CS and CS-MVP were prepared, and the synergic behavior of the mixed materials owing to inter- and intrachain interactions among hydroxy, methoxy, and amino groups was established. Inks with suitable viscosity values for 3D printing applications of the CS/CS-MVP blends in ratios of 60:40, 50:50, and 40:60 were prepared, and their viscosity values were assessed. Their prerequired shear thinning behavior has been long established, and the obtained inks are promising candidates for potential application in 3D printing procedures, along with drug-delivery systems. 

## Figures and Tables

**Table 1:** Degree of substitution of the samples CS-MVP 4-1, CS-MVP 2-1, CS-MVP 1-1, and CS-MVP 1-2.

Sample	Degree of Substitution (%)
CS-MVP 4-1	10.2
CS-MVP 2-1	13.6
CS-MVP 1-1	14.7
CS-MVP 1-2	15.4

**Table 2:** Contact angles of the samples CS, CS-MVP 4-1, CS-MVP 2-1, CS-MVP 1-1, and CS-MVP 1-2.

Sample	Contact Angle, θ (°)	
CS	74.5	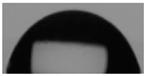
CS-MVP 4-1	73.5	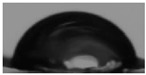
CS-MVP 2-1	67.8	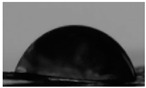
CS-MVP 1-1	58	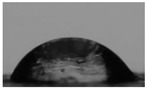
CS-MVP 1-2	82.6	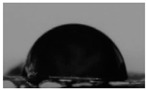

**Table 3:** Amount of the added MVP and ammonium persulfate quantities for the CS-MVP 4-1, 2-1, 1-1 and 1-2 ratios.

	CS (gr)	MVP (μL)	Ammonium Persulfate (mg)
CS-MVP 4-1	1	101	2
CS-MVP 2-1	1	203	2
CS-MVP 1-1	1	406	2
CS-MVP 1-2	1	812	2

## Data Availability

Data is contained within the article.
